# Clinical Features of Late-Life Depression with Different Neurobiochemical characteristics

**DOI:** 10.1192/j.eurpsy.2025.1355

**Published:** 2025-08-26

**Authors:** V. Pochueva, O. Yakovleva, T. Safarova

**Affiliations:** 1 FSBSI Mental Health Research Center, Moscow, Russian Federation

## Abstract

**Introduction:**

Depression is one of the most important medical and social problems in old age due to its high prevalence (10-25%) and a significant increase in the burden on social services and medical institutions. A diverse combination of biological factors of aging contributes to the polymorphism of clinical manifestations of late-life depression.

**Objectives:**

Study of the clinical features of depressed patients of late age of three identified neurobiochemical models of energy, antioxidant and glutamate metabolism.

**Methods:**

The study material consisted of 52 hospitalized patients (40 women and 12 men) aged 60-86 years with a depressive episode of recurrent depressive disorder, bipolar affective disorder and a single depressive episode (ICD-10). The patients were examined by clinical, psychometric, biochemical and statistical methods. Before starting therapy, psychometric assessments were performed using the Hamilton Anxiety and Depression Rating Scales and the Mini-Mental Status Examination. On the same day, the activity of enzymes of energy (cytochrome c oxidase - CO), antioxidant (glutathione reductase - GR and glutathione S-transferase - GST) and glutamate (glutamate dehydrogenase - GDH) metabolism enzymes in blood platelets was determined in patients.

**Results:**

In patients with a decrease in energy and antioxidant metabolism (↓GR, GST, HD and ↑GDH), there was a predominance of shallow apathetic depression of a “seasonal” nature with the presence of mild cognitive impairment, a later age of manifestation, and a high incidence of cerebrovascular pathology. Patients with “disharmonious” metabolism (↓ GR, GST, GD and ↑ CO) were characterized by an early onset of the disease, its longer duration, more severe and complex depression with a pronounced anxiety component. Patients with a conditionally “normal” metabolism were more likely to experience typical melancholy depression and the lowest incidence of severe cerebrovascular pathology.

**Conclusions:**

The relationship has been established between the clinical features of late-life depressions and changes in the activity of enzymes of energy, antioxidant and glutamate metabolism. It was revealed that the type of metabolism with “reduced” and “disharmonious” activity of these enzymes corresponds to the parameters of late and early manifesting depression. Thus, the clinical heterogeneity of late-life depression is closely related to different neurobiochemical types of metabolism.

**Disclosure of Interest:**

None Declared

